# Validity of a Consumer-Based Wearable to Measure Clinical Parameters in Patients With Chronic Obstructive Pulmonary Disease and Healthy Controls: Observational Study

**DOI:** 10.2196/56027

**Published:** 2024-11-06

**Authors:** Fien Hermans, Eva Arents, Astrid Blondeel, Wim Janssens, Nina Cardinaels, Patrick Calders, Thierry Troosters, Eric Derom, Heleen Demeyer

**Affiliations:** 1 Department of Rehabilitation Sciences, Ghent University, Corneel Heymanslaan 10, Entrance 46, Ghent, 9000, Belgium, 3293326915; 2 Department of Rehabilitation Sciences, KU Leuven, Leuven, Belgium; 3 Department of Chronic Diseases, Metabolism and Aging (CHROMETA) - BREATHE, KU Leuven, Leuven, Belgium; 4 Clinical Department of Respiratory Diseases, University Hospitals Leuven, Leuven, Belgium; 5 Clinical Department of Respiratory Medicine, Ghent University Hospital, Ghent, Belgium

**Keywords:** chronic obstructive pulmonary disease, COPD, wearable, Fitbit, clinical parameters, physical activity, validity, observational study, wrist-worn wearable, heart rate, heart rate variability, respiratory rate, oxygen saturation, devices, monitoring

## Abstract

**Background:**

Consumer-based wearables are becoming more popular and provide opportunities to track individual’s clinical parameters remotely. However, literature about their criterion and known-groups validity is scarce.

**Objective:**

This study aimed to assess the validity of the Fitbit Charge 4, a wrist-worn consumer-based wearable, to measure clinical parameters (ie, daily step count, resting heart rate [RHR], heart rate variability [HRV], respiratory rate [RR], and oxygen saturation) in patients with chronic obstructive pulmonary disease (COPD) and healthy controls in free-living conditions in Belgium by comparing it with medical-grade devices.

**Methods:**

Participants wore the Fitbit Charge 4 along with three medical-grade devices: (1) Dynaport MoveMonitor for 7 days, retrieving daily step count; (2) Polar H10 for 5 days, retrieving RHR, HRV, and RR; and (3) Nonin WristOX_2_ 3150 for 4 nights, retrieving oxygen saturation. Criterion validity was assessed by investigating the agreement between day-by-day measures of the Fitbit Charge 4 and the corresponding reference devices. Known-groups validity was assessed by comparing patients with COPD and healthy controls.

**Results:**

Data of 30 patients with COPD and 25 age- and gender-matched healthy controls resulted in good agreement between the Fitbit Charge 4 and the corresponding reference device for measuring daily step count (intraclass correlation coefficient [ICC_2,1_]=0.79 and ICC_2,1_=0.85, respectively), RHR (ICC_2,1_=0.80 and ICC_2,1_=0.79, respectively), and RR (ICC_2,1_=0.84 and ICC_2,1_=0.77, respectively). The agreement for HRV was moderate (healthy controls: ICC_2,1_=0.69) to strong (COPD: ICC_2,1_=0.87). The agreement in measuring oxygen saturation in patients with COPD was poor (ICC_2,1_=0.32). The Fitbit device overestimated the daily step count and underestimated HRV in both groups. While RHR and RR were overestimated in healthy controls, no difference was observed in patients with COPD. Oxygen saturation was overestimated in patients with COPD. The Fitbit Charge 4 detected significant differences in daily step count, RHR, and RR between patients with COPD and healthy controls, similar to those identified by the reference devices, supporting known-groups validity.

**Conclusions:**

Although the Fitbit Charge 4 shows mainly moderate to good agreement, measures of clinical parameters deviated from the reference devices, indicating that monitoring patients remotely and interpreting parameters requires caution. Differences in clinical parameters between patients with COPD and healthy controls that were measured by the reference devices were all detected by the Fitbit Charge 4.

## Introduction

In recent years, consumer-based wearables have become increasingly popular due to their relatively low cost, ease of use, and ability to provide real-time feedback on several clinical parameters such as heart rate and step count. In Europe, 26% of 45‐ to 74-year-olds used a wearable device in 2022 [1]. Alongside their rising popularity, wearable technologies also advanced significantly. Contemporary generation wrist-worn wearables use photoplethysmography technology, which measures the volumetric variations of blood circulation via an infrared light [2]. This technology enables to measure multiple parameters of autonomic function (ie, resting heart rate [RHR], heart rate variability [HRV], and respiratory rate [RR]) and oxygen saturation (SpO_2_) [3].

Chronic obstructive pulmonary disease (COPD) is the third leading cause of death worldwide [4]. Although COPD is a disease characterized by respiratory symptoms and exercise intolerance related to abnormalities of the airways, the alveoli, or both, COPD seems also to alter the autonomic nervous system [5]. The autonomic nervous system adjusts heart rate, blood pressure, and RR in response to internal and external stimuli [6]. Patients with COPD show an elevated RHR, a reduced HRV and an increased RR compared to healthy controls [5,7]. Besides the autonomic function, SpO_2_ is a clinical parameter that differs between patients with COPD and healthy controls, with patients with COPD having a lower SpO_2_ due to alterations in gas exchange and experiencing a sudden drop in SpO_2_ during exercise and when experiencing an acute exacerbation [4,8]. Both parameters of autonomic function and SpO_2_ are prognostic markers of mortality and thus important to monitor [5,8,9].

Wearables potentially enable continuous monitoring of clinical parameters remotely and unobtrusively over a long period of time [10]. Whereas manufacturers specify that consumer-based wearables are not intended for medical purposes as they do not qualify as medical devices, continuously monitoring these parameters would provide information on the management of the patients’ health at home, and investigate the effectiveness of interventions based on measures outside clinical visits in an easy way [11]. Moreover, given that these clinical parameters are linked to the worsening of health in various chronic diseases, it is tempting to actively monitor these parameters remotely, as this may lead to earlier detection of patients’ deterioration or provide an explanation for reduced engagement in physical activity [12-16].

Previous literature showed that Fitbit wearables are valid devices for monitoring daily step counts in healthy individuals and can be used to monitor patterns of physical activity in patients with COPD [17-19]. However, literature in the healthy population on the validity of Fitbit measuring other clinical parameters is scarce, and no data are available for patients with COPD [18,20-22]. These studies used various devices, including medical-grade devices (such as the ActiGraph GT3X+ and Dynaport MoveMonitor for daily step count, and the Polar H7 for heart rate), as well as gold-standard measurements (such as ECG Holter monitoring for heart rate), to assess the validity of Fitbit wearables.

The aim of this observational study was to investigate the criterion validity and the known-groups validity of a consumer-based wearable for monitoring physical activity (ie, daily step count) and parameters of autonomic function (ie, RHR, HRV, and RR) in patients with COPD and a reference population consisting of healthy age-matched controls. Furthermore, the criterion validity and known-groups validity of this consumer-based wearable for monitoring SpO_2_ was examined in patients with COPD.

## Methods

### Population and Design

This observational study was nested in a randomized controlled trial investigating the long-term effects of a telecoaching intervention in patients with COPD (NCT04139200). All patients with COPD included via Ghent University Hospital (Ghent, Belgium) and examined between April 2022 and June 2023 were enrolled in this substudy, ensuring representation of the entire patient cohort at this site. Patients aged 40 years and older with a smoking history of at least 10 pack years and with a clinical diagnosis of COPD (confirmed by spirometry [Tiffeneau-index<70%]) but no history of exacerbations in the past month were eligible to participate. Patients were excluded if they had orthopedic or other problems preventing them from improving physical activity, had undergone lung transplantation, were involved in a multidisciplinary rehabilitation program, or were unable to learn to work with electronic devices.

In addition, healthy controls were recruited between November 2022 and August 2023. These participants were 50‐80 years old, had never smoked or had stopped smoking more than 20 years before inclusion, had a Tiffeneau-index ≥70%, had no chronic health problems or orthopedic problems preventing them from being physically active, and did not participate in a rehabilitation program.

### Ethical Considerations

This study consisted of a single clinical visit and a follow-up period of 7 days. Both studies were approved by the ethical committee of Ghent University Hospital (BC-10267 [COPD] and ONZ-2022‐0387 [healthy controls]). All participants signed the informed consent prior to data collection and were assigned a study ID number. No compensation was provided for participation in this study.

### Clinical Assessments

At the clinical visit, the following assessments were performed in both groups: (1) sociodemographic and clinical data (age, sex, height, weight, smoking history, and medication intake); (2) postbronchodilator spirometry according to European Respiratory Society (ERS) – American Thoracic Society (ATS) guidelines [23]; (3) functional exercise capacity based on the best of two 6-minute walk tests following ERS-ATS guidelines [24]; and (4) Composite Autonomic Symptom Score (COMPASS) 31 questionnaire questioning autonomic nervous system symptoms [25]. In patients with COPD, the modified Medical Research Council scale (mMRC) questioning dyspnea and COPD Assessment Test (CAT) questioning health status were collected additionally [26,27].

During the 7-day wearing period following the clinical visit, participants were asked to wear a wrist-worn wearable, the Fitbit Charge 4, along with 3 approved medical-grade devices. During this wearing period, participants completed a daily diary including the time of waking up and going to bed, intake of medication, and consumption of beverages influencing the heart rate (eg, coffee, tea, alcohol, and energy drinks).

### Wearable

The Fitbit Charge 4 (Fitbit Inc) is a triaxial consumer-based wearable worn on the nondominant wrist of the participant. This device records daily step count (accelerometry), as well as RHR, HRV, RR, and SpO_2_ (photoplethysmography). These variables were extracted as day-by-day outcomes from the Fitbit platform, as calculated by the proprietary algorithms. Fitbit defines RHR as the heart rate while in a relaxed state during both sleep and being awake. HRV and RR are defined as the variation of duration between heartbeats, expressed as the root mean square of successive differences (RMSSD) in milliseconds (ms), and the number of breaths per minute during the night, respectively, when more than 3 hours of continuous sleep are recorded. Transcutaneous SpO_2_ was defined as the average hemoglobin SpO_2_ during the night (>3 hours of continuous sleep). Participants were instructed to wear the device for 7 consecutive days (24 hours per day). The device had a battery life of up to 7 days, but participants were advised to charge the battery when the battery level dropped below 10% and when they were not performing any activity.

### Reference Devices

#### Physical Activity—Dynaport MoveMonitor

The Dynaport MoveMonitor (DAM; McRoberts) is a triaxial accelerometer validated to objectively measure physical activity in patients with COPD and healthy controls [28,29]. Participants were instructed to wear the monitor at the lower back during waking hours for 7 consecutive days, except for bathing and water activities, according to current recommendations [30]. Days with a wearing time lower than 8 hours were excluded for further analyses [30]. Wearing time and daily step count were extracted from the monitor for further analysis.

#### Cardiac Autonomic Function—Polar H10

The Polar H10 sensor chest strap (Polar Electro Oy) is a validated device to capture heart rate and HRV in healthy subjects [31]. This sensor was moistened before being applied below the chest muscles of the participants, as described by the manufacturer. To collect heart rate data for 100 hours, participants wore a corresponding Polar Ignite 2 watch (at the preferred wrist), which was used as a Polar H10 data logger. They were instructed to wear these devices for 5 consecutive days (24 hours per day), except for bathing and water activities, based on recommendations and the maximum recording capacity of 100 hours. Data were recorded using a 1-second time interval. Heart rate and beat-to-beat RR intervals (time between 2 successive R-waves of the QRS signal on the electrocardiogram) were extracted from the device using the Polar Flow web service.

To obtain the RHR, the average heart rate during the night (actual sleep time, ie, starting 1 hour later than the reported time of going to bed and ending 1 hour earlier than the reported time of waking up) was calculated to best match Fitbit’s definition. Nights with 3 hours of sleep or less were excluded for further analyses.

The beat-to-beat RR intervals (time between 2 successive R-waves of the QRS signal on the electrocardiogram) were transferred to Kubios HRV Software (version 4.0.1, Kuopio) to analyze HRV (expressed as RMSSD in ms) and RR [32]. The actual sleep time as reported in the diary was entered into the software. Nights with 3 hours of sleep or less were excluded for further analyses to best fit with Fitbit’s definition.

#### SpO_2_ (COPD Only)—Nonin WristOX_2_ 3150

The wearable finger pulse oximeter Nonin WristOX_2_ 3150 (Nonin Medical Inc) was used as a reference device for measuring SpO_2_, as it has become a commonly used device for home monitoring of patients with COPD and has a high accuracy (±2%) according to the manufacturer [33]. Patients with COPD were instructed to wear the device on the index finger of their dominant hand for 4 consecutive nights (limited battery life of 48 hours using continuous measurements, allowing up to 12 hours of monitoring per night). Data were recorded using a 4-second time interval. Data were stored on the internal memory of the device and downloaded after the 7-day follow-up period using the nVISION software (version 6.5.1.2, Nonin Medical Inc). The average SpO_2_ during the night measured by Nonin was calculated after removing impossible values. Nights with 3 hours of sleep (as judged by the Fitbit device) or less were excluded for further analyses to best fit with Fitbit’s definition.

### Statistical Analyses

The sample size of this substudy was chosen to align with prior research within this field [18]. All statistical analyses were performed using the SAS statistical package (version 9.4, SAS Institute). Data are presented as mean (SD) or median (IQR), as appropriate after testing for normality using the Shapiro-Wilk test. Statistical significance was set at *P*<.05 for all analyses.

Criterion validity was investigated by comparing day-by-day data obtained by the Fitbit Charge 4 with the reference devices by use of a 2-tailed paired *t* test, Bland-Altman plots, and intraclass correlation coefficients (ICC_2,1_). The cutoffs that were used to interpret the findings were ICC<0.50 as “poor,” ICC=0.50‐0.75 as “moderate,” ICC=0.75‐0.90 as “good,” and ICC>0.90 as “excellent” [34]. These analyses were performed for patients with COPD and healthy controls separately. To evaluate whether the Fitbit Charge 4 is able to pick up day-by-day fluctuations, the delta (day minus day-1) of each measured clinical parameter was calculated on consecutive days. The agreement between the Fitbit Charge 4 and the corresponding reference device with regard to day-by-day fluctuations was determined via Pearson correlation. The correlation was interpreted using the cutoffs *r*<0.30 classed as “no correlation,” *r*=0.30‐0.50 as “weak correlation,” *r*=0.50‐0.70 as “moderate correlation,” *r*=0.70‐0.90 as “strong correlation,” and *r*>0.90 as “very strong correlation” [35]. Next, known-groups validity was assessed by investigating whether the differences between patients with COPD and healthy controls were picked up by the reference device and the Fitbit Charge 4 in the same way. For this, the outcomes were compared between patients with COPD and healthy controls for both devices using an unpaired *t* test. The differences between both groups were examined by an interaction effect based on a mixed model analysis. As a sensitivity analysis, all analyses were performed with the exclusion of patients taking beta-blockers.

## Results

### Patient Characteristics

In total, 32 patients with COPD and 26 age- and gender-matched healthy controls were included in this study. Overall, valid data of 30 patients with COPD (1 patient dropped out and 1 patient was not willing to wear the additional devices) and 25 healthy controls (1 participant was excluded because spirometry displayed an obstructive syndrome) were obtained, but across the clinical parameters, there is a variation in participants included in the analyses. This variation can be attributed to various reasons, such as technical problems (the Fitbit not capturing clinical parameters at night, sudden interruptions in the measurement by the reference device, battery issues), as well as participants forgetting to wear the (reference) device. Baseline characteristics are shown in Table 1.

**Table 1.** Characteristics of all participants included in the analyses.

	Patients with COPD^a^ (n=30)	Healthy controls (n=25)	*P* value
Age (years), mean (SD)	70 (7)	68 (7)	.28
Sex (male), n (%)	23 (77)	20 (80)	.77
BMI (kg/m^2^), mean (SD)	27 (6)	28 (4)	.86
Forced expiratory volume in the first second (% predicted), mean (SD)	54 (15)	109 (15)	<.001
**Severity of COPD, n (%) **			
I-II	2 (7)-15 (50)	N/A^b^	N/A
III-IV	12 (40)-1 (3)	N/A	N/A
Use of β-blockers, n (%)	8 (27)	4 (16)	.34
Current smokers, n (%)	1 (3)	0 (0)	.36
6MWD^c^ (m), mean (SD)	482 (95)	618 (81)	<.001
6MWD (% predicted), mean (SD)	78 (14)	97 (10)	<.001
COMPASS 31^d^ (score), median (IQR)	11 (4‐21)	5 (2-12)	.005
mMRC^e^ (score), median (IQR)	2 (1-2)	N/A	N/A
CAT^f^ (score), mean (SD)	15 (6)	N/A	N/A

a COPD: chronic obstructive pulmonary disease.

b N/A: not applicable.

c 6MWD: 6-minute walking distance.

d COMPASS 31: Composite Autonomic Symptom Score 31 questionnaire (0‐100); a higher score indicated more symptoms of autonomic dysfunction.

e mMRC: modified Medical Research Council dyspnea scale (0‐4); a higher score indicated more dyspnea.

f CAT: COPD Assessment Test (0‐40); a higher score indicated a worse health status.

### Criterion Validity

#### Physical Activity

Daily step count was analyzed based on 199 (min-max 4 to 7 days per patient, n=30) and 157 (4 to 7 days per participant, n=24) overlapping data points for patients with COPD and healthy controls, respectively. As expected, patients with COPD were less active compared to healthy controls (Table 2).

**Table 2.** Average step count, resting heart rate, heart rate variability, and respiratory rate in patients with COPD^a^ and healthy controls.

	Patients with COPD (n=30), mean (SD)	Healthy controls (n=25), mean (SD)	Difference, mean (SD)	*P* value^b^
**Daily steps (steps/day** **)**				
Dynaport MoveMonitor	5625 (3618)	7933 (4263)	−2308 (3915)	<.001
Fitbit Charge 4	7423 (4325)	9055 (4956)	−1632 (4613)	.001
**Resting heart rate (beats/min** **)**				
Polar H10	70 (8)	60 (10)	10 (9)	<.001
Fitbit Charge 4	70 (8)	64 (10)	6 (9)	<.001
**Heart rate variability (ms)**				
Polar H10	27 (19)	33 (21)	−6 (20)	.16
Fitbit Charge 4	24 (14)	26 (13)	−2 (14)	.53
**Respiratory rate (breaths/min** **)**				
Polar H10	16 (2)	14 (2)	2 (2)	<.001
Fitbit Charge 4	16 (3)	15 (2)	1 (2)	.01

a COPD: chronic obstructive pulmonary disease.

b *P* values based on unpaired *t* test.

**Figure 1.**
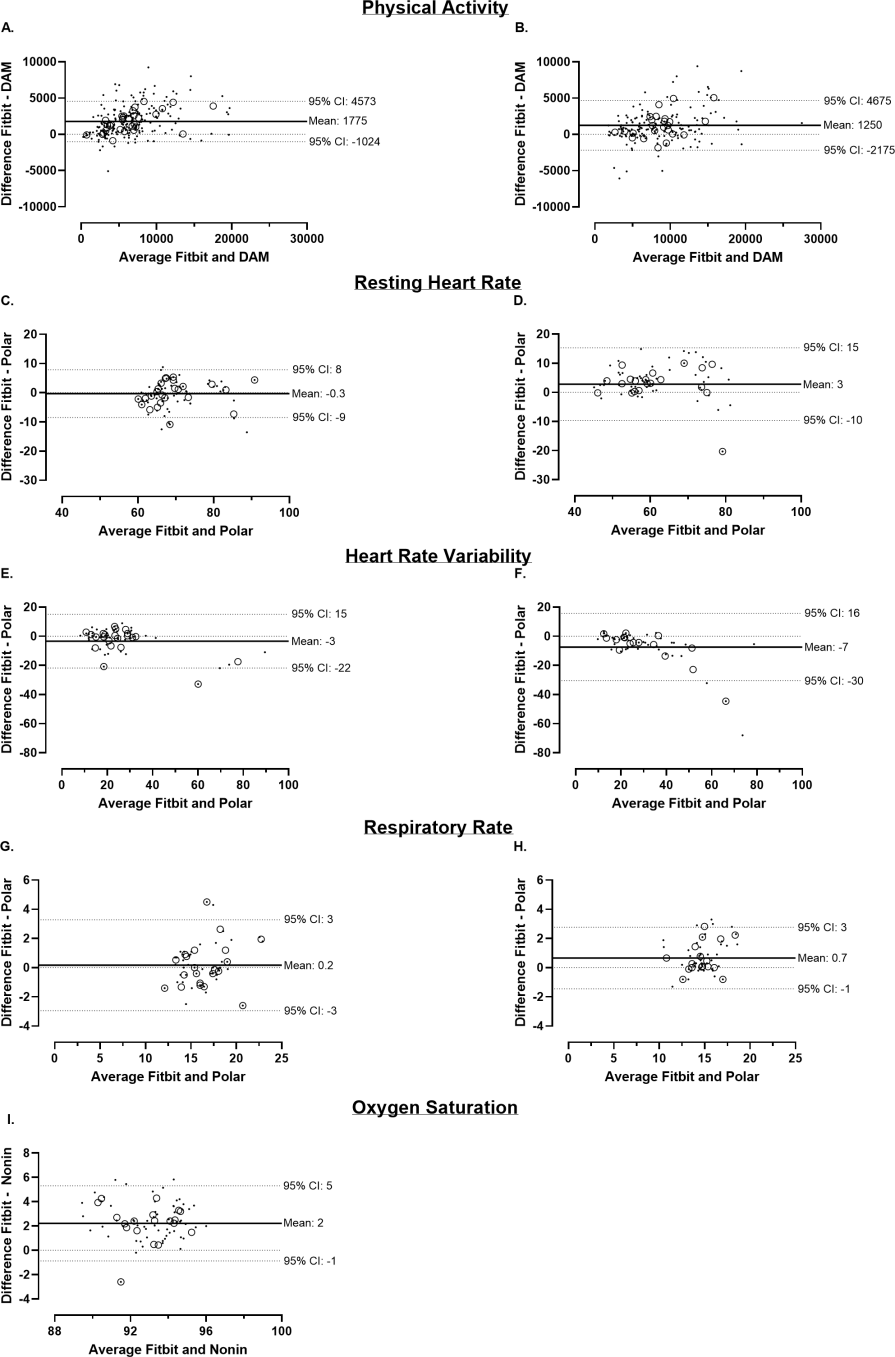
Bland-Altman plots with mean and 95% CI for Fitbit Charge 4 compared to the reference devices. (**A, B**) Daily steps (steps/day) measured by Fitbit Charge 4 and DAM (COPD: n=30 and healthy controls: n=24). (**C, D**) Resting heart rate (beats/min) measured by Fitbit Charge 4 and Polar H10 (COPD: n=25 and healthy controls: n=20). (**E, F**) Heart rate variability (RMSSD in ms) measured by Fitbit Charge 4 and Polar H10 (COPD: n=22 and healthy controls: n=16). (**G, H**) Respiratory rate (breaths/min) measured by Fitbit Charge 4 and Polar H10 (COPD: n=21 and healthy controls: n=17). (**I**) Oxygen saturation (%) measured by Fitbit Charge 4 and Nonin WristOX_2_ 3150 (COPD: n=19). Large open dots represent the mean individual outcome per patient. Small dots represent daily data. Mean and 95% CI are calculated based on average data. COPD: chronic obstructive pulmonary disease; DAM: Dynaport MoveMonitor; RMSSD: root mean square of successive differences.

In both groups, the mean step count measured by the Fitbit Charge 4 was significantly higher compared to DAM (mean, SD; COPD: ∆+1798, SD 2070 steps/day; *P*<.001 and healthy controls: ∆+1122, SD 2297 steps/day; *P*<.001). However, in both groups, a good agreement between the devices was found (COPD: ICC_2,1_=0.79; 95% CI 0.36‐0.90 and healthy controls: ICC_2,1_=0.85; 95% CI 0.74‐0.91). These findings are supported by the Bland-Altman plots depicted in Figure 1A and 1B. Concurrent validity with associated scatterplots can be found in Multimedia Appendix 1.

**Figure 2.**
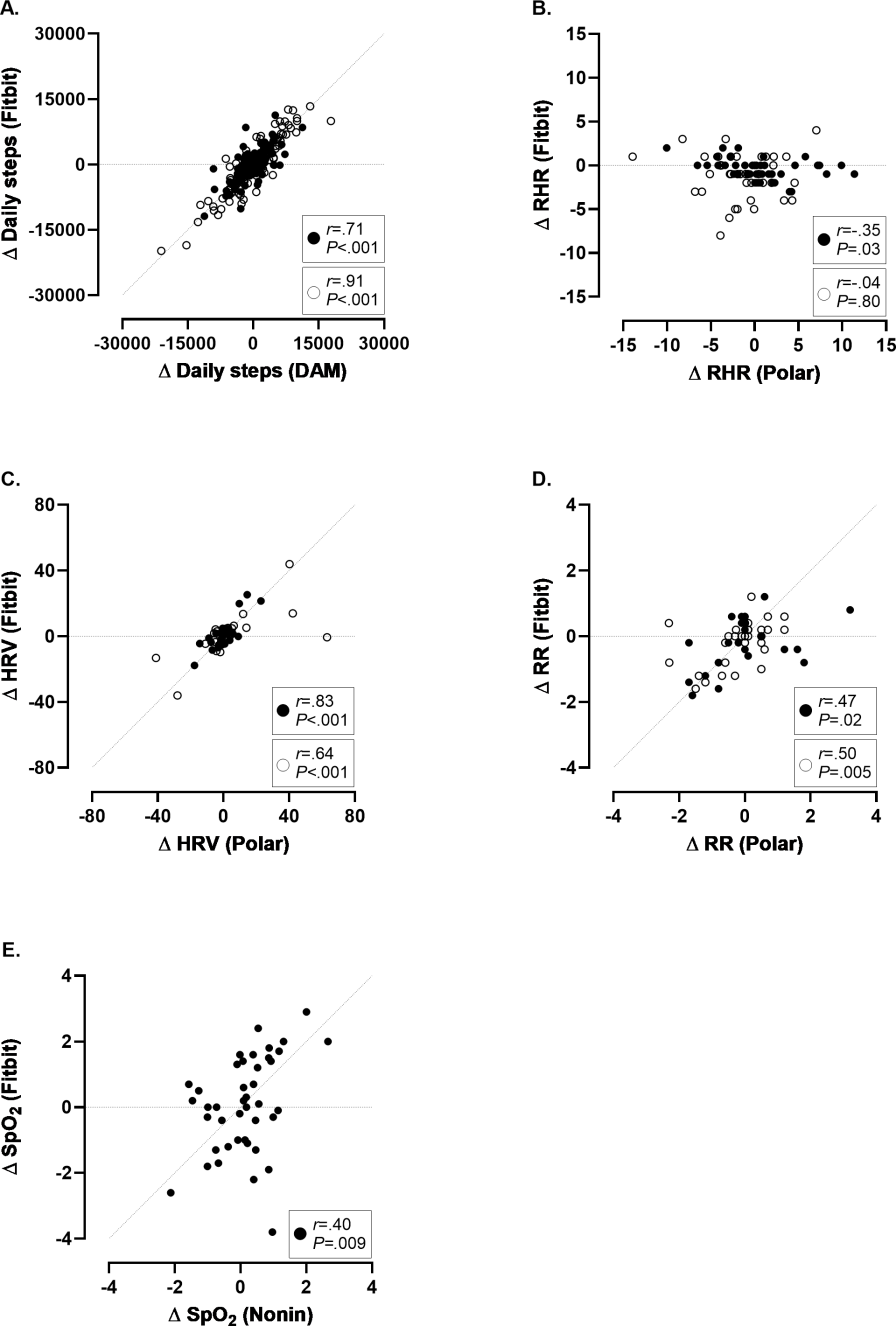
Scatterplots of the day-by-day fluctuations in daily step count, parameters of autonomic function, and SpO_2_ in patients with COPD and healthy controls. (**A**) Day-by-day fluctuations in daily step count (COPD: 169 data points and healthy controls: 133 data points). (**B**) Day-by-day fluctuations in resting heart rate (COPD: 42 data points and healthy controls: 39 data points). (**C**) Day-by-day fluctuations in heart rate variability (COPD: 27 data points and healthy controls: 28 data points). (**D**) Day-by-day fluctuations in respiratory rate (COPD: 24 data points and healthy controls: 30 data points). (**E**) Day-by-day fluctuations in oxygen saturation (COPD: 42 data points). Patients with COPD are depicted in the closed dots and healthy controls are depicted in the open dots. An identity line is displayed on the scatterplots. COPD: chronic obstructive pulmonary disease; DAM: Dynaport MoveMonitor; HRV: heart rate variability; RHR: resting heart rate; RR: respiratory rate; SpO_2_: oxygen saturation.

Figure 2A shows the scatterplot of the day-by-day fluctuations in daily step count measured with the Fitbit Charge 4 and the DAM. A strong to very strong association was observed between both devices in patients with COPD (*r*=0.71) and healthy controls (*r*=0.91), respectively.

#### Resting Heart Rate

RHR was analyzed based on 66 (min-max 1 to 4 days per patient, n=25) and 59 (1 to 4 days per healthy control, n=20) overlapping data points. RHR was higher in patients with COPD compared to healthy controls (Table 2).

In the COPD group, RHR measured by the Fitbit showed no difference compared to Polar H10 (∆−0.3, SD 5 beats/min; *P*=.67), whereas Fitbit significantly overestimated RHR in the healthy control group (∆+4, SD 6 beats/min; *P*<.001). In both groups, Fitbit Charge 4 and Polar H10 showed a good agreement for assessing RHR (COPD: ICC_2,1_=0.80; 95% CI 0.70‐0.87 and healthy controls: ICC_2,1_=0.79; 95% CI 0.50‐0.90). The findings are depicted in Bland-Altman plots (Figure 1C and 1D). A strong association between the devices was observed in both groups (Multimedia Appendix 1).

A weak negative association was observed in patients with COPD for picking up day-by-day fluctuations in RHR (*r*=−0.35; Figure 2B).

#### Heart Rate Variability

HRV analyses are based on 49 (min-max 1 to 3 days per patient, n=22) and 44 (1 to 4 days per participant, n=16) overlapping data points for patients with COPD and healthy controls, respectively. No difference in HRV was observed between patients with COPD and healthy controls (Table 2).

The Fitbit Charge 4 significantly underestimated HRV in both groups (respectively in COPD and healthy controls: ∆−3, SD 8 ms; *P*=.03 and ∆−7, SD 13 ms; *P*=.001). In patients with COPD, a good agreement was observed between both devices (ICC_2,1_=0.87; 95% CI 0.77‐0.93). Whereas in the healthy control group, a moderate agreement was found for measuring HRV (ICC_2,1_=0.69; 95% CI 0.43‐0.83). Bland-Altman plots (Figure 1E and 1F) represent these results. Scatterplots showing the concurrent validity can be found in Multimedia Appendix 1.

In patients with COPD, a strong association was found for picking up day-by-day fluctuations in HRV (*r*=0.83), whereas healthy controls exhibited a moderate association (*r*=0.64; Figure 2C).

#### Respiratory Rate

RR was analyzed based on 45 (min-max 1 to 5 days per patient, n=21) and 47 (1 to 4 days per healthy control, n=17) overlapping data points. RR was higher in patients with COPD compared to healthy controls (Table 2).

In patients with COPD, no difference was detected in RR measured by Fitbit Charge 4 compared to Polar H10 (∆+0.3, SD 1 breaths/min; *P*=.25). However, in the healthy control group, the Fitbit Charge 4 significantly overestimated the RR (∆+1, SD 1 breaths/min; *P*<.001). A good agreement was observed in patients with COPD, as well as healthy controls (COPD: ICC_2,1_=0.84; 95% CI 0.72‐0.91 and healthy controls: ICC_2,1_=0.77; 95% CI 0.44‐0.89). These findings are supported by the Bland-Altman plots depicted in Figure 1G and 1H. In both groups, a strong association was found (Multimedia Appendix 1).

A weak association was observed in both groups for picking up day-by-day fluctuations in RR (COPD: *r*=0.47 and healthy controls: *r*=0.50; Figure 2D).

#### Oxygen Saturation

SpO_2_ was analyzed based on 61 (min-max 1 to 5 days per patient, n=19) overlapping data points in patients with COPD. Fitbit Charge 4 significantly overestimated SpO_2_ (∆+2, SD 2%; *P*<.001). A poor agreement between both devices was found (ICC_2,1_=0.32; 95% CI −0.10‐0.65). The Bland-Altman analysis is shown in Figure 1I.

Figure 2E displays the scatterplot of the day-by-day fluctuations measured with the Fitbit Charge 4 and the Nonin WristOX_2_ 3150, showing a weak association for SpO_2_ in patients with COPD (*r*=0.40).

### Known-Groups Validity

The significant differences between patients with COPD and healthy controls in daily steps, RHR, and RR identified by the reference devices were picked up in a similar way by the Fitbit Charge 4 (see Table 2). The difference between both groups is significantly smaller when assessing RHR or RR using the Fitbit Charge 4 compared to the reference device (*P*<.05).

### Sensitivity Analysis

Excluding participants on stable doses of beta-blockers had minimal impact on the results. The agreement between the Fitbit Charge 4 and its corresponding reference devices remained unchanged. The exclusion of participants taking beta-blockers had no effect on the known-groups validity (Multimedia Appendix 2).

## Discussion

### Principal Findings

This study, which aimed to investigate the validity of a consumer-based wearable, the Fitbit Charge 4, in patients with COPD and healthy controls found mixed results for criterion validity. First, the Fitbit Charge 4 significantly overestimated daily step count and significantly underestimated HRV in patients with COPD and healthy controls. In patients with COPD, RHR and RR are not different between the Fitbit Charge 4 and the Polar H10, but both parameters were overestimated in the healthy control group. The Fitbit Charge 4 significantly overestimated SpO_2_ in patients with COPD. Second, the agreement between the Fitbit Charge 4 and the corresponding reference devices is moderate to good for monitoring most clinical parameters (ie, daily steps, RHR, HRV, and RR) but poor for tracking SpO_2_. The Fitbit is able to pick up day-by-day fluctuations in daily step count and HRV but lacks accuracy to pick up the small day-by-day fluctuations in RHR, RR, and SpO_2_. The known-groups validity of the Fitbit Charge 4 is good. All expected differences between patients with COPD and age- and gender-matched healthy controls are picked up by the wearable.

Our results are consistent with previous research in older adults in free-living conditions showing that the daily step count measured by the Fitbit Charge, a wrist-worn wearable, is highly correlated with the daily step count measured by a validated accelerometer, but the Fitbit Charge significantly overestimates the daily step count [21,36,37]. Previous studies conducted in other patient cohorts within our department showed that the wrist-worn Fitbit device significantly overestimated the daily step count in healthy individuals and cancer survivors, whereas it did not in people with Parkinson disease [38,39]. Blondeel et al [19] concluded that the Fitbit Alta, also a wrist-worn wearable, did not significantly overestimate step count in patients with COPD, but did in healthy controls. This corresponds well with our findings, although in our sample, the wearable also overestimated daily step count in patients with COPD. This could potentially be explained by the inclusion of patients with a better functional exercise tolerance (6-minute walking distance of 482 m vs 454 m) in this study.

A few studies examined the validity of Fitbit measuring the heart rate during sleep among healthy adults in different situations (ie, home environment and laboratory-based setting). One study in healthy adolescents showed that Fitbit significantly underestimated the heart rate by 0.9 beats/min on average as compared to ECG, while another study in healthy adults found no difference between Fitbit and the reference device [40,41]. These findings are consistent with our results in the COPD group but are inconsistent with our results showing that Fitbit significantly overestimates RHR by 4 beats/min in the healthy control group. The discrepancy in results can possibly be attributed to the difference in how the definition is applied, with Fitbit defining RHR as the heart rate while in a relaxed state during both sleep and being awake, whereas we calculated RHR from Polar for sleep time values only because we do not have exact information on how RHR is calculated by Fitbit.

To the best of our knowledge, the validity of Fitbit measuring HRV, RR, and SpO_2_ has not been investigated so far. However, some research has been performed regarding other wearables also using photoplethysmography technology (eg, Apple watch, Garmin, and Polar), showing a large range for measuring HRV (ICC ranging from 0.24 to 0.99) depending on the wearable used in a laboratory setting in healthy adults [42]. These results are consistent with our findings in healthy controls (ICC_2,1_=0.69). Interestingly, we found a higher agreement in patients with COPD (ICC_2,1_=0.87), but no previous research has been conducted on the validity of wearables measuring HRV among this population.

Data on the validity of a wrist-worn wearable estimating RR based on photoplethysmography have not been published so far. Existing studies have only focused on the validity of devices measuring this parameter using different technologies (ie, wearable biosensors and wearable pressure sensors) [43,44]. This difference in technology makes it challenging to compare these results.

Several studies indicate that the Apple Watch Series 6 shows a good agreement for measuring SpO_2_ compared to the gold-standard (ie, arterial blood gas analyses) or reference devices (ie, oximeters) in patients with lung diseases (ICCs ranging from 0.90 to 0.94), as well as healthy individuals (mean bias 1.7, SD 2.1%) [45-47]. These results are in contrast with our findings achieving an ICC of 0.32. The results of Garmin concerning the measurements of SpO_2_ in healthy individuals vary widely, with ICCs ranging from 0.28 to 0.55, which is more in line with our results [48,49]. In our study, the SpO_2_ was measured during sleep, where the contact between the watch and the skin was maybe occasionally suboptimal due to unintentionally lying on the wearable, leading to divergent measurements through photoplethysmography. This is in contrast to the other studies where the measurement of SpO_2_ was conducted under controlled settings over a short period of time (maximum 10 minutes) while being awake.

Regarding the known-groups validity, the differences in daily step count (lower in COPD), RHR (higher in COPD), and RR (higher in COPD) were in line with previous research [7,9,19]. We could not confirm a difference in HRV, which was shown to be lower in COPD in previous research (COPD and healthy controls: 27, SD 19 ms and 33, SD 21 ms; *P*=.16 [this study] vs 11, SD 3 ms and 19, SD 7 ms; *P*=.002, respectively) [50]. This study confirms that previously laboratory-based findings are now also observed in free-living situations.

It is remarkable that our findings indicate that Fitbit estimates certain clinical parameters, such as RHR, HRV, and RR, better in patients with COPD as compared to healthy controls. In this regard, we speculate that faster aging of the skin and skin thinning, a typical feature in patients with COPD on inhaled and systemic steroids, improve reflection of lights for measuring clinical parameters through photoplethysmography compared to slightly thicker skins in healthy controls as the distance of the infrared light to measure volumetric changes in the blood is reduced [51-53].

### Clinical Implications

While our results suggest that Fitbit can measure certain clinical parameters better in patients with COPD than in healthy controls, it is not the best choice if one aims to measure important parameters of autonomic function (ie, RHR and RR) as well as SpO_2_ in patients with COPD, as the wearable fails to pick up day-by-day fluctuations. Nevertheless, Fitbit can be used for commercial purposes and well-being monitoring, including data on daily step counts and HRV. Advanced and accurate (medical) devices are more appropriate for remotely monitoring clinical parameters. However, these results are only based on a 5‐ to 7-day assessment in a stable situation. Longer time series of data also including larger fluctuations in the clinical parameters (eg, when patients experience a deterioration in their health) are needed to confirm these findings.

### Strengths and Limitations

This study offers valuable insights into the validity of a popular wearable device to estimate various clinical parameters during a free-living situation. Our approach involved studying both patients with COPD, as well as age- and gender-matched healthy controls, aiming to establish evidence in both populations. Even though 4 of the 25 (16%) control participants were using beta-blockers, they can be considered as a representative sample of the general population [54]. Nonetheless, this study has some limitations. First, it should be mentioned that Fitbit operates as a black box, using proprietary algorithms of which its details are not disclosed. This makes it challenging to establish an agreement with reference devices. Second, technical issues arose, preventing the collection of certain data. In some cases, Fitbit failed to measure parameters of autonomic function or SpO_2_, rendering a comparison with reference devices impossible. Third, validated medical-grade devices that show good agreement with gold-standard methods were used in this study to assess the validity of the Fitbit Charge 4 in free-living conditions. Unfortunately, gold-standard devices (ie, video recording or manual step counting [physical activity] and ECG Holter monitoring [HRV]) are restricted to laboratory-based measurements. However, it is important to acknowledge that the reference devices may still involve measurement inaccuracies. Given that the measurements were conducted in daily life, the results have a high level of generalizability. Fourth, a selection bias within our healthy controls is difficult to avoid, as probably the more motivated individuals are more prone to participate in these kinds of studies. However, the clinical parameters in our control group align with those reported in the general population [55,56].

### Conclusions

Both in patients with COPD and healthy controls, measures of clinical parameters collected by the commercially Fitbit Charge 4 showed moderate to good agreement with the reference devices. However, these measures deviated significantly. In patients with COPD, the Fitbit Charge 4 is accurate in measuring RHR and RR. The wearable lacks accuracy to pick up day-by-day fluctuations in RHR, RR, and SpO_2_; hence, the Fitbit Charge 4 should be used with caution when information on clinical parameters is collected over a short period of time.

## Supplementary material

10.2196/56027 Multimedia Appendix 1 Concurrent validity between the Fitbit Charge 4 and the corresponding reference device for measuring daily step count, resting heart rate, heart rate variability, and respiratory rate.

10.2196/56027 Multimedia Appendix 2 Sensitivity analysis—criterion and known-groups validity of the Fitbit Charge 4 in participants not taking beta-blockers.

## Abbreviations

ATS: American Thoracic Society
CAT: COPD Assessment Test
COMPASS: Composite Autonomic Symptom Score
COPD: chronic obstructive pulmonary disease
DAM: Dynaport MoveMonitor
ERS: European Respiratory Society
HRV: heart rate variability
ICC: intraclass correlation coefficient
mMRC: modified Medical Research Council
RHR: resting heart rate
RMSSD: root mean square of successive differences
RR: respiratory rate
SpO_2_: oxygen saturation
