# Supplementary material for: Validity of a Consumer-Based Wearable to Measure Clinical Parameters in Patients With Chronic Obstructive Pulmonary Disease and Healthy Controls: Observational Study
Source: JMIR Mhealth Uhealth. 2024 Nov 6;12:e56027. doi: 10.2196/56027 (PMC11559788; doi:10.2196/56027)
Supplement: Multimedia Appendix 1 [file mhealth-v12-e56027-s001.docx]

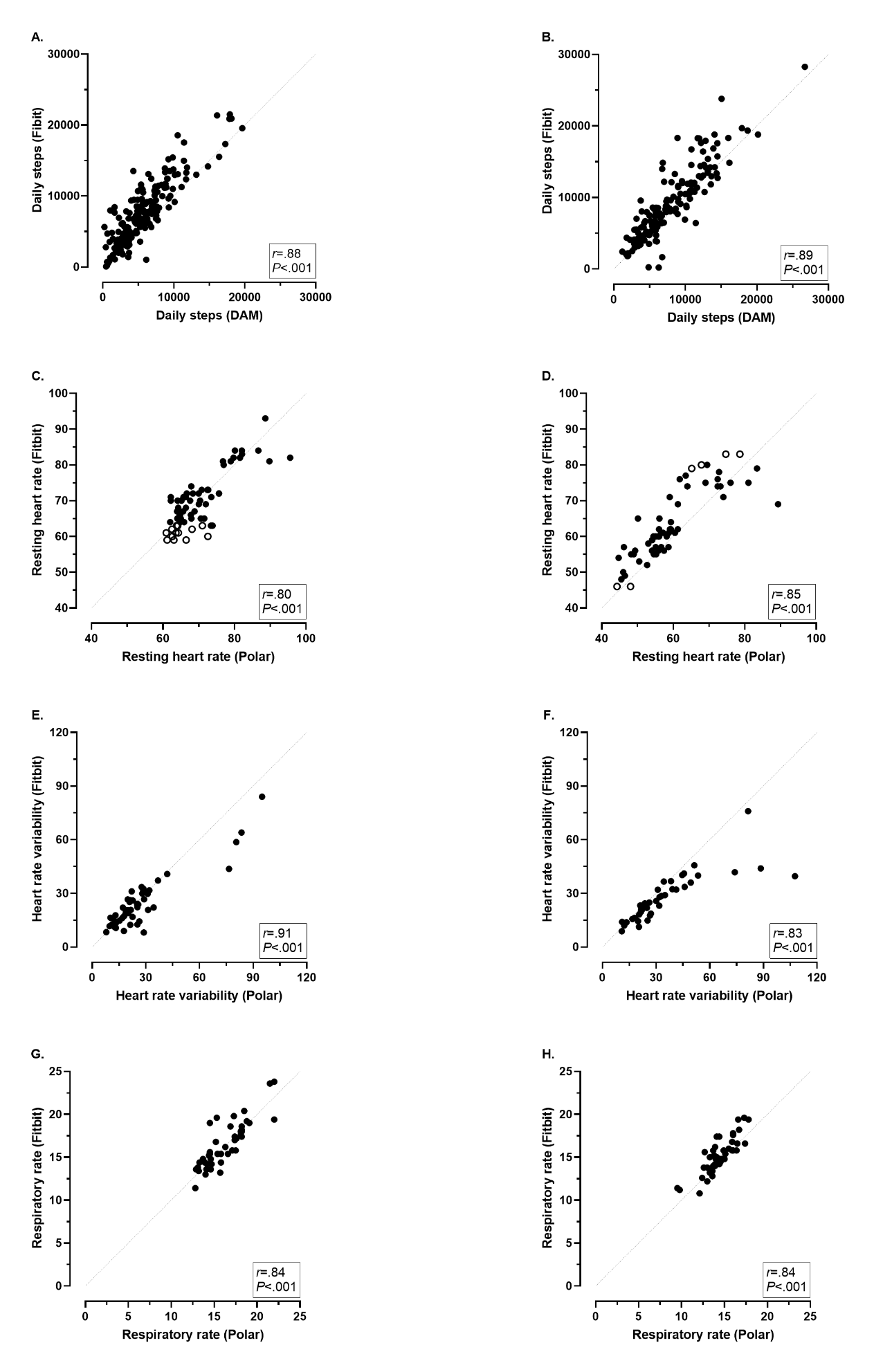


**Figure S1:** **Scatter plots of the concurrent validity between the Fitbit Charge 4 and the corresponding reference device via Pearson correlation.** (A, B) Daily steps measured by Fitbit Charge 4 and DAM (COPD: 199 datapoints and healthy controls: 157 datapoints), (C, D) Resting heart rate measured by Fitbit Charge 4 and Polar H10 (COPD: 66 datapoints and healthy controls: 59 datapoints), (E, F) Heart rate variability measured by Fitbit Charge 4 and Polar H10 (COPD: 49 datapoints and healthy controls: 44 datapoints), (G, H) Respiratory rate measured by Fitbit Charge 4 and Polar H10 (COPD: 45 datapoints and healthy controls: 47 datapoints). Patients taking beta-blockers are depicted in the open dots. Identity line is displayed on the scatter plots. DAM= Dynaport MoveMonitor; r= Pearson correlation.
