# Supplementary material for: Validity of a Consumer-Based Wearable to Measure Clinical Parameters in Patients With Chronic Obstructive Pulmonary Disease and Healthy Controls: Observational Study
Source: JMIR Mhealth Uhealth. 2024 Nov 6;12:e56027. doi: 10.2196/56027 (PMC11559788; doi:10.2196/56027)
Supplement: Multimedia Appendix 2 [file mhealth-v12-e56027-s002.docx]

*Table S1*

|  | **Patients with COPD  (N = 22)** | | | | **Healthy controls  (N = 21)** | | | |
| --- | --- | --- | --- | --- | --- | --- | --- | --- |
|  | **ICC_2,1_** | **(95% CI)** | **r** | **p-value** | **ICC_2,1_** | **(95% CI)** | **r** | **p-value** |
| Daily steps | 0.85 | (0.52-0.93) | .91 | <.001 | 0.84 | (0.73-0.90) | .87 | <.001 |
| RHR | 0.80 | (0.69-0.88) | .81 | <.001 | 0.77 | (0.49-0.88) | .83 | <.001 |
| HRV | 0.89 | (0.79-0.94) | .93 | <.001 | 0.69 | (0.43-0.84) | .83 | <.001 |
| RR | 0.84 | (0.71-0.91) | .85 | <.001 | 0.83 | (0.58-0.92) | .88 | <.001 |
| SpO_2_ | 0.28 | (-0.09-0.63) | .64 | <.001 |  |  |  |  |

**Table S1: Intraclass correlation coefficients with 95% confidence interval and Pearson correlation for the different clinical parameters in patients with COPD and healthy controls** with exclusion of participants taking beta-blockers**.** RHR= Resting heart rate; HRV= Heart rate variability; RR= Respiratory rate; SpO_2_= Oxygen saturation; ICC**_2,1_**= Intraclass Correlation Coefficients; CI= Confidence interval; r= Pearson correlation.

*Table S2*

|  | **Patients with COPD**  **(N = 22)** | **Healthy controls**  **(N = 21)** | **Difference** | **p-value** |
| --- | --- | --- | --- | --- |
| **Daily steps (steps/day)** | | | | |
| DAM | 5575 (3851) | 7832 (4050) | -2257 (3947) | <.001 |
| Fitbit Charge 4 | 7075 (4456) | 8843 (4646) | -1768 (4547) | .001 |
| **Resting heart rate (beats/min)** | |  |  |  |
| Polar H10 | 71 (8) | 59 (10) | 12 (9) | <.001 |
| Fitbit Charge 4 | 72 (7) | 63 (9) | 9 (8) | <.001 |
| **Heart rate variability (msec)** | |  |  |  |
| Polar H10 | 27 (20) | 33 (23) | -6 (5) | .25 |
| Fitbit Charge 4 | 24 (15) | 26 (14) | -2 (3) | .61 |
| **Respiratory rate (breaths/min)** | |  |  |  |
| Polar H10 | 16 (3) | 14 (2) | 2 (2) | <.001 |
| Fitbit Charge 4 | 17 (3) | 15 (2) | 2 (3) | .007 |

**Table S2: Average step count, resting heart rate, heart rate variability and respiratory rate in patients with COPD and healthy controls** with exclusion of participants taking beta-blockers, presented as mean (standard deviation). P-value based on unpaired t-test.
